# sLORETA current source density analysis of evoked potentials for spatial updating in a virtual navigation task

**DOI:** 10.3389/fnbeh.2014.00066

**Published:** 2014-03-04

**Authors:** Hai M. Nguyen, Jumpei Matsumoto, Anh H. Tran, Taketoshi Ono, Hisao Nishijo

**Affiliations:** System Emotional Science, Graduate School of Medicine and Pharmaceutical Sciences, University of ToyamaToyama, Japan

**Keywords:** virtual navigation, ERPs, current source density, place recognition, spatial updating

## Abstract

Previous studies have reported that multiple brain regions are activated during spatial navigation. However, it is unclear whether these activated brain regions are specifically associated with spatial updating or whether some regions are recruited for parallel cognitive processes. The present study aimed to localize current sources of event related potentials (ERPs) associated with spatial updating specifically. In the control phase of the experiment, electroencephalograms (EEGs) were recorded while subjects sequentially traced 10 blue checkpoints on the streets of a virtual town, which were sequentially connected by a green line, by manipulating a joystick. In the test phase of the experiment, the checkpoints and green line were not indicated. Instead, a tone was presented when the subjects entered the reference points where they were then required to trace the 10 invisible spatial reference points corresponding to the checkpoints. The vertex-positive ERPs with latencies of approximately 340 ms from the moment when the subjects entered the unmarked reference points were significantly larger in the test than in the control phases. Current source density analysis of the ERPs by standardized low-resolution brain electromagnetic tomography (sLORETA) indicated activation of brain regions in the test phase that are associated with place and landmark recognition (entorhinal cortex/hippocampus, parahippocampal and retrosplenial cortices, fusiform, and lingual gyri), detecting self-motion (posterior cingulate and posterior insular cortices), motor planning (superior frontal gyrus, including the medial frontal cortex), and regions that process spatial attention (inferior parietal lobule). The present results provide the first identification of the current sources of ERPs associated with spatial updating, and suggest that multiple systems are active in parallel during spatial updating.

## Introduction

The ability to navigate one's environment is a fundamental survival skill, required to locate sources of food (e.g., restaurants) and other important resources, such as shelter, and simply to navigate between desired locations. Spatial updating enables the navigator to keep track of the spatial relationship between themself and their surroundings when moving. According to the types of information being used in spatial updating, navigations can be classified as either piloting (landmark-based navigation) or path integration (dead reckoning or velocity-based navigation) (Gallistel, 1990; Yoder et al., 2011). In piloting, the navigator updates his or her current position and orients within the environment by using external cues, such as significant landmarks (specific buildings, intersections, etc.), in conjunction with a map. In path integration, the navigator integrates self-motion information (e.g., velocity and acceleration information) to estimate his or her current position and orientation relative to the starting point (Gallistel, 1990; Etienne, 1992). Self-motion (ideothetic) information is derived from the integration of vestibular information from the otoliths and semicircular canals, proprioceptive information from the muscles, tendons, and joints, motor efferent copies, and optical flow. Recent studies suggest that optical flow provides sufficient information for updating position and orientation (Riecke et al., 2002; Gramann et al., 2005).

Thus, spatial updating allows topographical orientation, which is generally defined as an individual's ability to orient and navigate from one place to another in the environment (Maguire et al., 1996). Spatial navigation requires many complex cognitive processes, such as attention, perception, memory, and decision-making skills (Redish, 1999; Brunsdon et al., 2007). Visual mental imagery, in particular, has been suggested to be a cognitive skill critical for successfully navigating in the environment (Farah, 1989; Riddoch and Humphreys, 1989; Davis and Coltheart, 1999; Brunsdon et al., 2007). During actual spatial navigation, individuals usually use mental imagery to internally represent spatial information, such as landmarks and routes, and use this information to navigate the environment (Farah, 1989; Davis and Coltheart, 1999; Brunsdon et al., 2007). In this way, individuals create a mental image of the environment in which they are navigating and to manipulate and rotate their spatial map to update their current position with respect to their target location (Palermo et al., 2008). Furthermore, neuropsychological studies of patients with brain damage or congenital neurodevelopmental defects suggest that compromised topographical orientation abilities are associated with disturbances in the capacity to form mental images of pathways and landmarks that would be encountered during navigation (De Renzi, 1982; Aguirre and D'Esposito, 1999; Iaria et al., 2005). These findings suggest that internal representations of the environment, and manipulation of these representations, are indispensable cognitive functions required for spatial navigation.

Recent noninvasive studies that simulate spatial navigation using virtual reality and photos of scenes have identified the brain regions recruited during spatial navigation: the hippocampus, parahippocampal gyrus, posterior cingulate gyrus, temporal cortex, insula, superior and inferior parietal cortex, precuneus, dorsolateral prefrontal cortex, medial prefrontal cortex, premotor area, and supplemental motor area, etc. (Aguirre and D'Esposito, 1997; Aguirre et al., 1998; Maguire et al., 1998; Burgess et al., 2001; Hartley et al., 2003; MacEvoy and Epstein, 2007; Spiers and Maguire, 2007a,b,c; Wolbers et al., 2007; Iseki et al., 2008). Because navigation induces activation of many cortical regions simultaneously, activity in these areas must be integrated and functionally interrelated. Consistent with this idea, parallel coherent activation has been reported during virtual navigation (Li et al., 2009; Hori et al., 2013).

However, it is unknown if the above activated brain regions are associated with spatial updating or with other cognitive processes; no fMRI studies investigated brain activity at the moment when subjects explicitly updated their spatial locations due to low temporal resolution. Although three previous electroencephalogram (EEG) studies investigated spatial updating (Bellebaum and Daum, 2006; Peterburs et al., 2011, 2013), these studies investigated updating of retinal coordinates of images after saccades, but not updating of own locations. The aim of the present study was to record EEGs while the subjects explicitly updated their spatial locations during virtual navigation. To this end, we have set up two task conditions; the control phase of the task required no spatial updating since green lines on the floor indicated the path, while the test phase of the task without the green lines required explicit spatial updating based on relationships among multiple landmarks in the virtual space. In the test phase, beep sounds, which were generated at the moment when they successfully reached the spatial reference points, indicated that they were located at the correct places. In the control phase, the same beep sounds were generated when the subjects reached the same spatial locations although spatial updating was not required. In this study, event-related potentials (ERPs) in response to the beep sounds generated at the moment subjects reached spatial reference points and updated their locations in a virtual environment were recorded. The current source density of ERPs components was analyzed by the standardized low-resolution brain electromagnetic tomography (sLORETA) method (Pascual-Marqui, 2002), and compared between the two task conditions.

Furthermore, recent studies suggest different theories: (1) Wang and Spelke (2002) suggest that egocentric spatial representation dominates, wherein the subject is in the center of the reference frame coordinates, whereas (2) Burgess (2006) suggests that both egocentric and allocentric (the center of the reference frame is independent of the subject) representations are processed in parallel during updating and navigation. These differences in spatial representation might underlie individual differences in navigation strategies [e.g., allocentric (bird-view) or egocentric (landmark) strategies] (Jordan et al., 2004). The results by sLORETA are discussed in terms of these two forms of spatial representation.

## Materials and methods

### Subjects

Twelve healthy right-handed male university subjects (mean age, 23.3 ± 0.69 years) participated in the study. They were naïve to the task used in the present study, and none of the subjects had a history of neurological problems. All subjects were treated in strict compliance with the Declaration of Helsinki and the U.S. Code of Federal Regulations for the protection of human participants. The experiments were conducted with the full consent of each participant using a protocol approved by the ethical committee at the University of Toyama. The subjects had no previous experience with participation in similar experiments.

### Experimental paradigms

**Figure 1:**
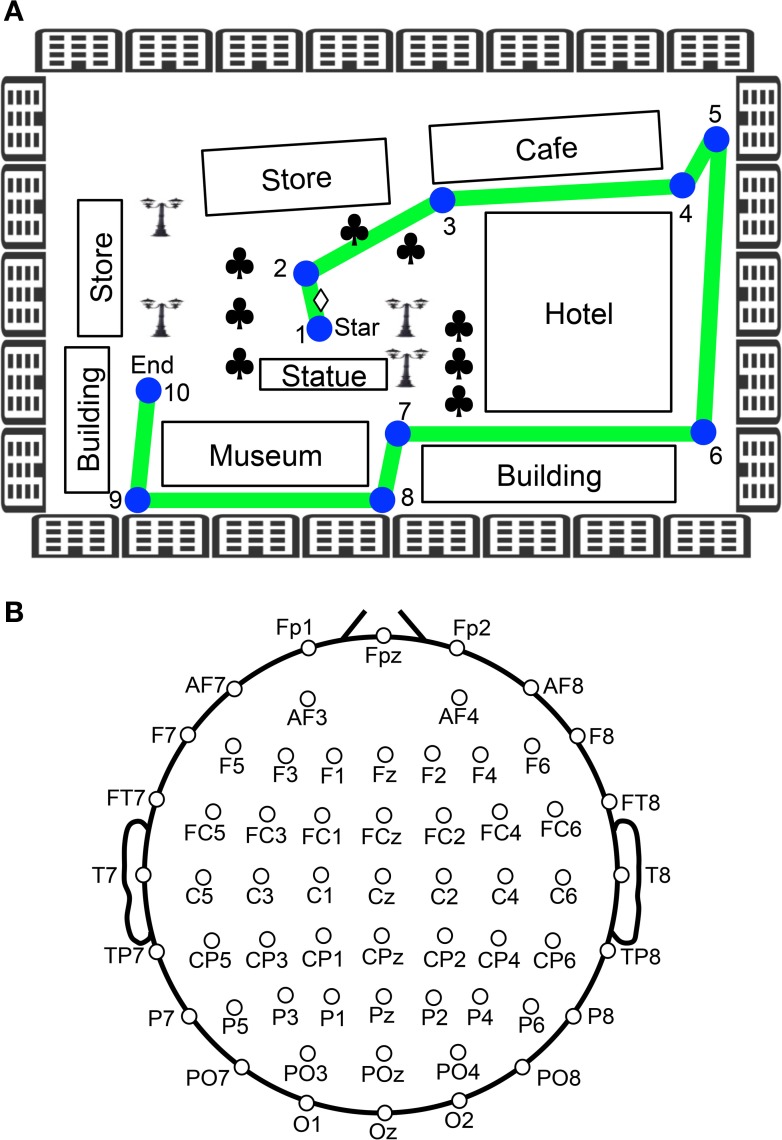
**Experimental paradigm of the virtual navigation task. (A)** Navigation route in the virtual town. In the control phase, 10 spatial reference points (checkpoints) were blue-colored circles labeled with numbers, which were connected by a green line. When the task was initiated subjects were always located at the position indicated by a diamond between checkpoints 1 and 2. Subjects were then required to sequentially trace the checkpoints from 1 to 10. In the test phase, the subjects were required to perform the same task three times, except that the 10 circular checkpoints and green line were made invisible in the virtual town. Subjects performed the task while undergoing EEG recording. Clovers indicate trees. **(B)** Arrangement of the electrodes.

The subjects were seated 1 m from a 20-inch LCD monitor in a chair that was grounded, within a dimly lit, shielded, room. For this task, a large virtual town was created using commercial 3D software (EON Studio ver.2.5.2, EON Reality Inc., Irvine, CA, USA). The virtual town consisted of streets and a series of buildings (Figure 1A). The subjects were required to manipulate a joystick with their right hand in order to navigate the virtual town presented on the monitor from a 3D first-person view. They grasped the joystick using their thumb, forefinger, and middle finger in a pronated hand position, and could move the joystick in all directions at a constant speed. The distance travelled by the joystick was a maximum of 2.5 cm from the center position in any direction, which corresponded to rotation of the joystick from a perpendicular line by 30°. Participants were able to freely navigate at constant speed in the forward, backward, right, and left directions using the joystick.

After setting up the electrodes, the subjects were given three trials to learn the navigation route and the layout of the virtual town. The navigation route contained 10 circular checkpoints labeled with numbers from 1 to 10, which were sequentially connected by a green line on the streets (Figure 1A). The subjects were required to sequentially trace the checkpoints from 1 to 10 along the green line by manipulating the joystick (control phase). When subjects entered each correct checkpoint, a beep sound lasting 0.53 s was generated. When the subjects entered checkpoint 10, the task was terminated. After a 1-min inter-trial interval, the next trial began by displaying a scene near checkpoint 1 in the virtual town. After these three learning trials (control phase), the subjects were required to perform the same task three times, except that the 10 circular checkpoints and green line were not shown in the virtual town (test phase). However, the same beep sound was generated when they reached each checkpoint. EEG recordings were performed throughout the control and test phases of the experiment. EMG recordings were performed in the test phases of the experiment.

### Recordings

The EEG (bandpass filtered at 0.3–120 Hz, with a sampling rate of 500 Hz) was recorded from 60 Ag/AgCl electrodes that were mounted on the subject's scalp, based on the International 10–20 extended system (Figure 1B). These were referenced to the average reference, and impedance was maintained below 5 kΩ. Electrooculograms (EOGs) with the same bandpass and sampling rate were also recorded to detect blinking and eye movements. A ground electrode was placed on the forehead.

### Data analysis

The EEG data were processed using Matlab (V7.10.4) (The Math Works, Natick, MA, USA) with the EEGLAB toolbox (Delorme and Makeig, 2004) before the data were analyzed by sLORETA. EEG artifacts due to the task (i.e., eye blink and saccade-related artifacts) were removed by independent component analysis (ICA) (Makeig et al., 1997, 1999; Jung et al., 2000; Delorme and Makeig, 2004). Short epochs including an EEG signal exceeding ±100 μV were also discarded from the data. To analyze the evoked potentials (ERPs) generated when the subjects arrived at the checkpoints (control phase) or spatial reference points corresponding to the checkpoints (test phase), 2 s of EEG data were extracted, 1 s before and 1 s after entering each checkpoint or spatial reference point.

The ERPs were then analyzed by the sLORETA software (Pascual-Marqui, 2002) (http://www.uzh.ch/keyinst/loreta.htm) to estimate the current source density. Briefly, sLORETA calculates the standardized current source density at each of the 6239 voxels in the gray matter and the hippocampus of the MNI-reference brain. This calculation of the current source density is based upon a linear weighted sum of the scalp electric potentials. sLORETA estimates the underlying sources under the assumption that the neighboring voxels should have a maximally similar electrical activity. Current source densities in each voxel between two conditions were compared by permutation test on paired data. For this comparison, sLORETA software performs “non-parametric randomization” of the data (see Nichols and Holmes, 2002, for a detailed description of permutation test theory). Therefore, the method is non-parametric, and computes the empirical probability distribution, and does not rely nor needs normality. Since multiple cerebral cortical areas and hippocampus were activated during navigation (see Introduction), the cerebral cortical areas including the hippocampus were determined as regions of interest before sLoreta analysis was performed.

All the data are expressed as mean ± s.e.m. All statistical significance was set at *P* < 0.05. Statistical analyses of EPR amplitudes and task duration were performed with a commercial statistical package, the Statistical Package for the Social Sciences (SPSS, Ver. 19; SPSS Inc. Chicago, IL).

## Results

### Behavioral results

**Figure 2:**
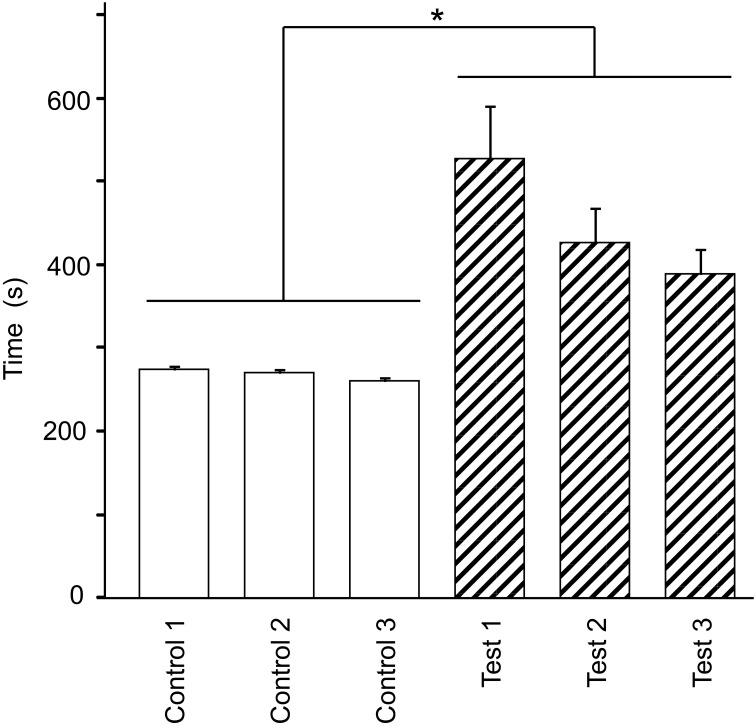
**Effect of eliminating the guiding green path on the duration spent on the virtual navigation task**. Asterisks indicate significant differences (*P* < 0.05).

Figure 2 shows the mean time required to traverse 10 checkpoints across the three control trials and the mean time required to traverse the 10 spatial reference points across the three test trials. There were significant differences in the time that elapsed among the six trials [repeated-measures one-way ANOVA with Greenhouse-Geisser correction; *F*_(1.936, 21.292)_ = 12.262, *P* = 0.003]. *Post-hoc* tests indicated that elapsed time was significantly increased in the test phase (Bonferroni test, *P* < 0.05). These results suggest that cognitive demand was larger in the test than in the control phases.

**Figure 3:**
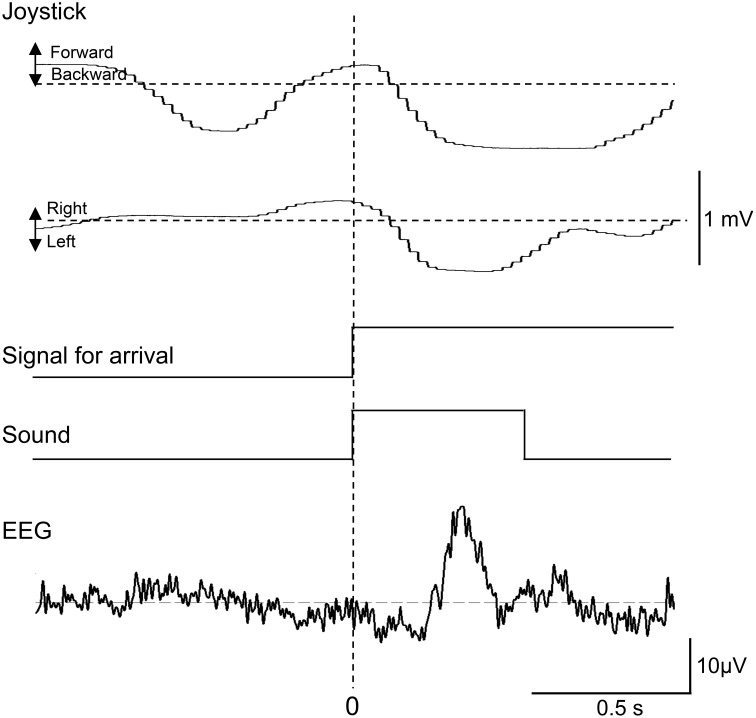
**Representative recordings of joystick movements, EEGs, and event signals from one subject upon entering a spatial reference point in the test phase**. Signal for arrival indicates arrival at the spatial reference point.

Representative recordings of joystick movements and EEGs from one subject in the test phase are shown in Figure 3. The subject manipulated the joystick to approach the spatial reference points. When the subject entered the spatial reference point at time zero, the beep sound was generated. In response to arriving at the spatial reference point, positive potentials peaking at a latency of around 340 ms were observed.

### Evoked potentials

**Figure 4:**
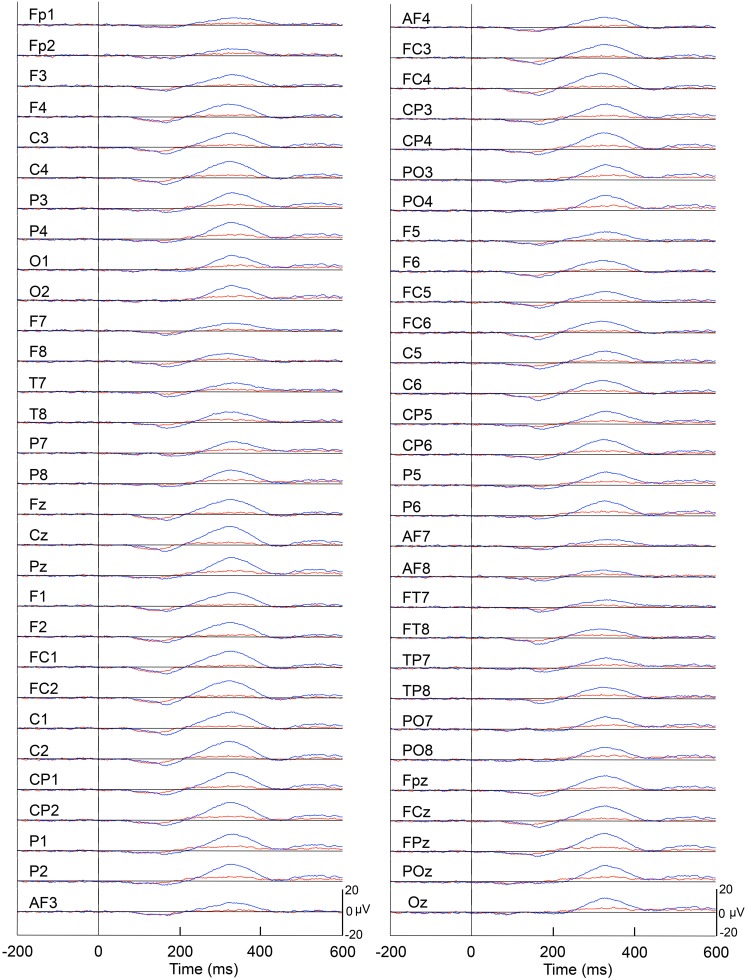
**Averaged ERPs with vertex-positivity in the control and test phases**. Blue and red recordings indicate averaged ERPs in the test and control phases, respectively. Zero in the time scale indicates arrival at the spatial reference point.

**Figure 5:**
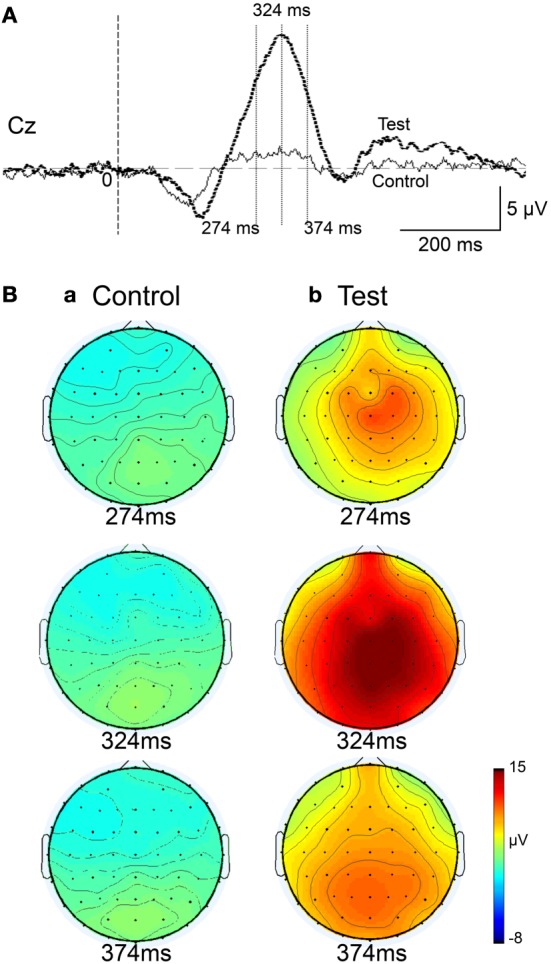
**Topographical maps of the averaged ERPs at latencies surrounding the ERP peak latency**. Latencies of examined ERPs are shown in **(A)**. The three topographical maps are indicated in **(B)**: three topographical maps of the ERPs in the control **(a)** and test **(b)** phases.

**Figure 6:**
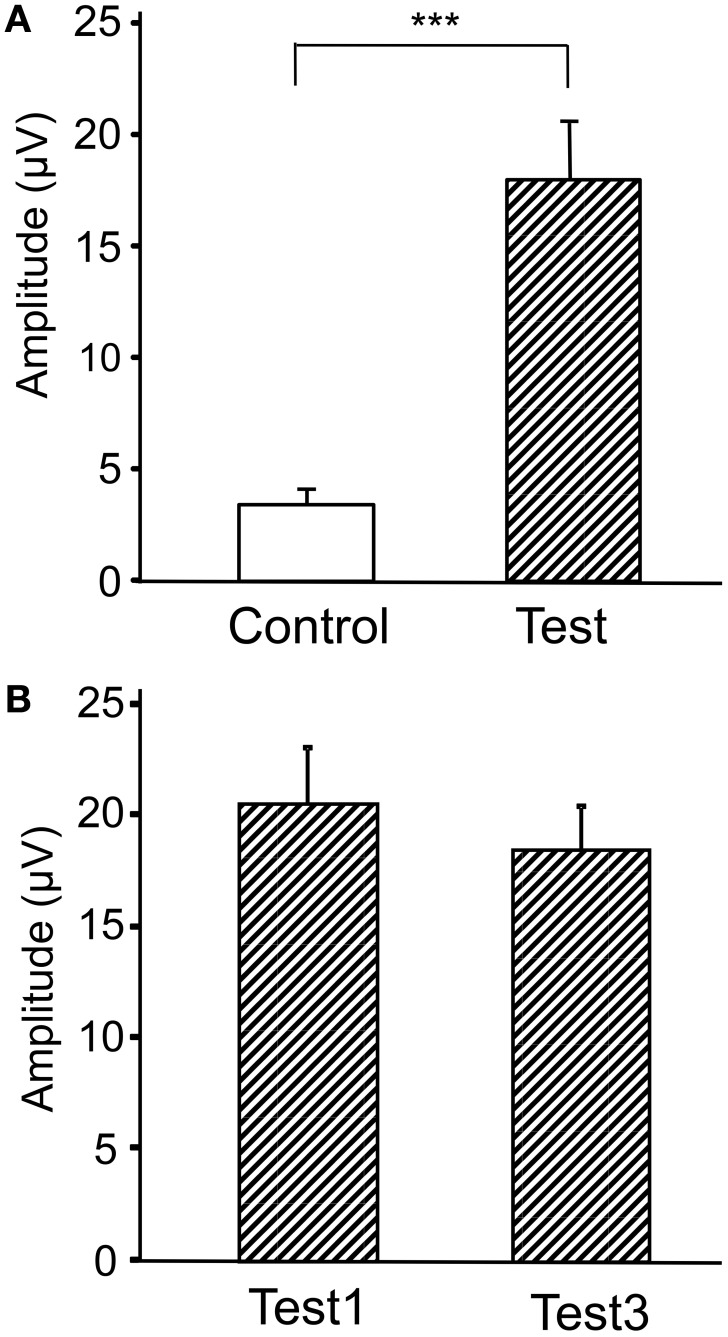
**Comparison of the peak amplitudes of the averaged ERPs between the control and test phases (A) and between the first and third trials in the test phase (B)**. ^***^p < 0.001.

**Figure 7:**
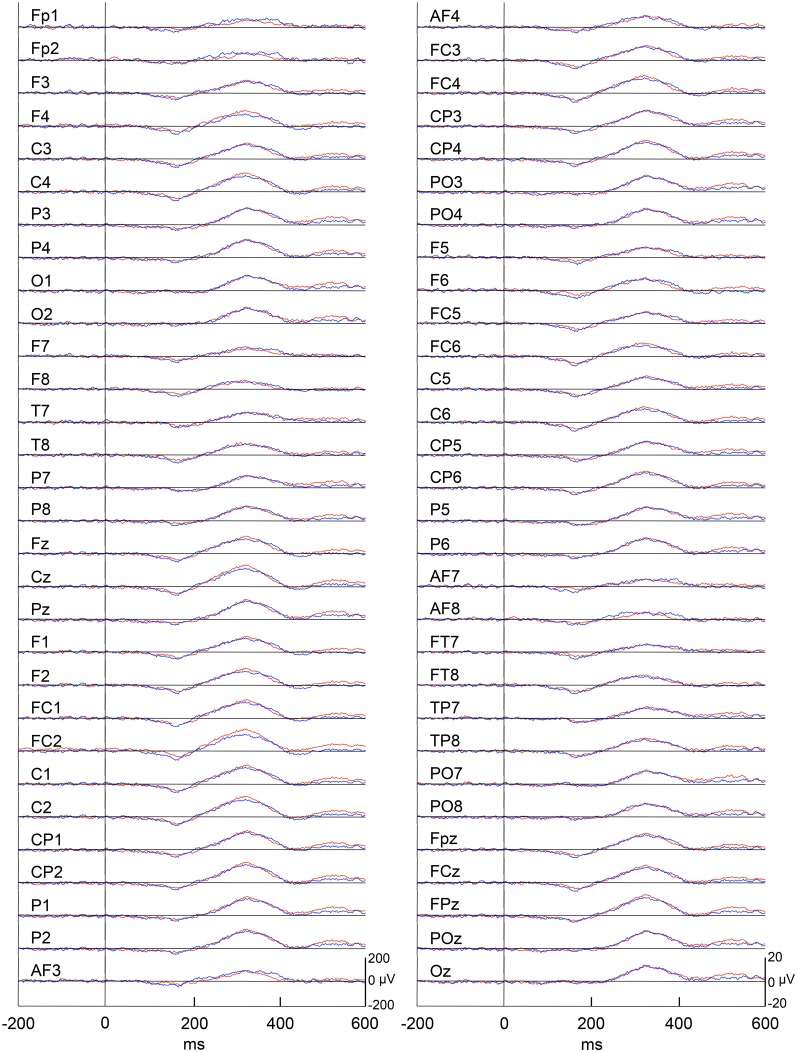
**Averaged ERPs with vertex-positivity in the test phase**. Blue and red recordings indicate averaged ERPs in the first and third trials in the test phase, respectively. Zero in time scale indicates arrival at the spatial reference points.

Figure 4 represents averaged ERPs aligned with the arrival at the checkpoints and spatial reference points. In the test phase, more prominent positive waveforms (blue traces) were observed in the fronto-parieto-occipital area compared to the control phase (red traces). Figure 5 shows topographical maps of the vertex-positive ERPs at 274 (50 ms before the peak latency)-, 324 (peak latency)-, and 374 (50 ms after the peak latency)-ms latencies around the peak. In the test phase, larger vertex-positive ERPs were observed compared with the control phase. Figure 6A shows a comparison between the peak amplitudes of the ERPs in Cz between the control and the test phases. Statistical comparison indicated that the peak amplitudes were significantly larger in the test phase relative to the control phase (paired *t*-test, *P* < 0.001). Figure 7 represents averaged ERPs in the first (red traces) and third (blue trials) trials. Almost identical waveforms were observed in both the trials. Figure 6B shows the comparison of the peak amplitudes of the ERPs in Cz between the first and third trials in the test phase. Statistical comparison indicated that there were no significant differences in the peak amplitudes between the first and third trials in the test phase (paired *t*-test, *P* > 0.05). These results indicate that these ERPs were not simply novelty-induced potentials.

### Current source localization of the evoked potentials

**Figure 8:**
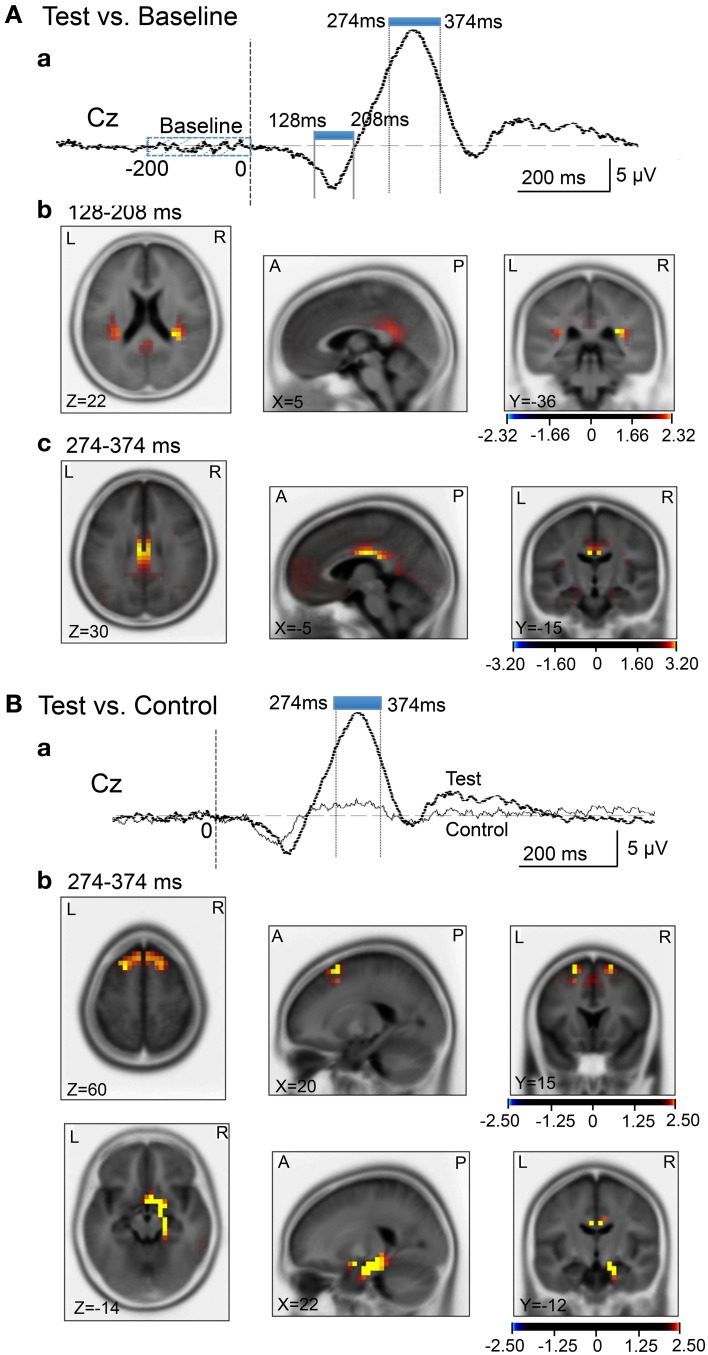
**sLORETA statistical nonparametric maps comparing the current source density in the baseline before arrival at the spatial reference points and ERPs after arrival (A), and comparing the current source density in the ERPs between the control and test phases (B)**. **(A)** Significant increase in current source density in the ERPs compared with the baseline at the 128- to 208-ms **(b)** and the 274- to 374-ms **(c)** latencies; **(a)** analyzed time windows indicated by blue thick lines. **(B)** Significant increase in current source density of the ERPs in the test phase compared with the control phase at the 274- to 374-ms **(b)** latency; **(a)** analyzed time windows indicated by blue thick lines. Calibration bars indicate *t*-values.

We analyzed current source density of the ERPs with the early negative (128–208 ms) and late positive (274–374 ms) peaks. First, we compared the current source densities of the ERPs upon arrival at the spatial reference points in the test phase to the current source densities at baseline, before entering the spatial reference points (Figure 8A). In the 128- to 208-ms latency **(Aa)**, current source density of the initial negative deflection was significantly higher in the posterior cingulate cortex, retrosplenial cortex, and bilateral posterior insula cortex **(Ab)**. In the 274- to 374-ms latency, current source density of the vertex-positive ERPs was significantly higher in the posterior cingulate and retrosplenial cortices **(Ac)**.

Second, we compared the current source densities of the ERPs between the test and control phases (Figure 8B). In the 274- to 374-ms latency **(Ba)**, current source density in the test phase was significantly higher in the superior frontal gyrus (area 6) including the medial frontal cortex **(Bb)**. Furthermore, current source density was significantly higher in the right entorhinal cortex/hippocampus, parahippocampal cortex, and posterior cingulate cortex (**Figure 8Bb**).

**Figure 9:**
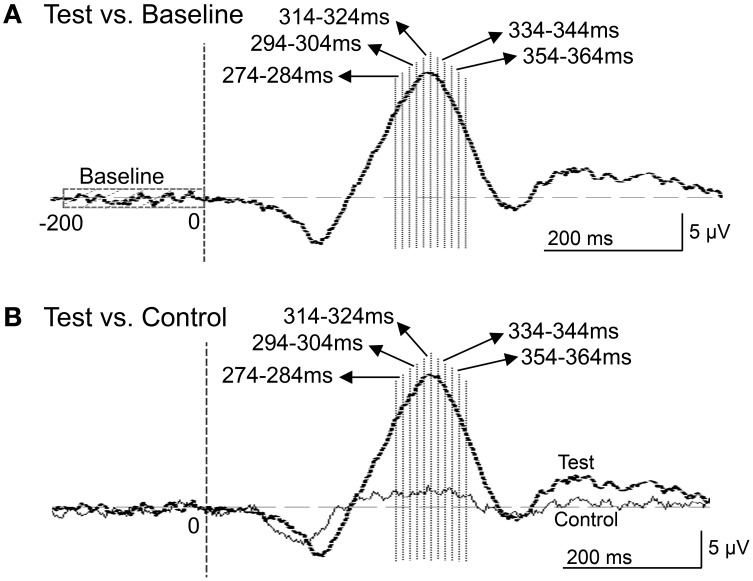
**Five 10-ms time windows for sLORETA analyses in the comparison between the baseline and ERPs in the test phase (A) and the comparison between the control and test phases (B)**.

**Figure 10:**
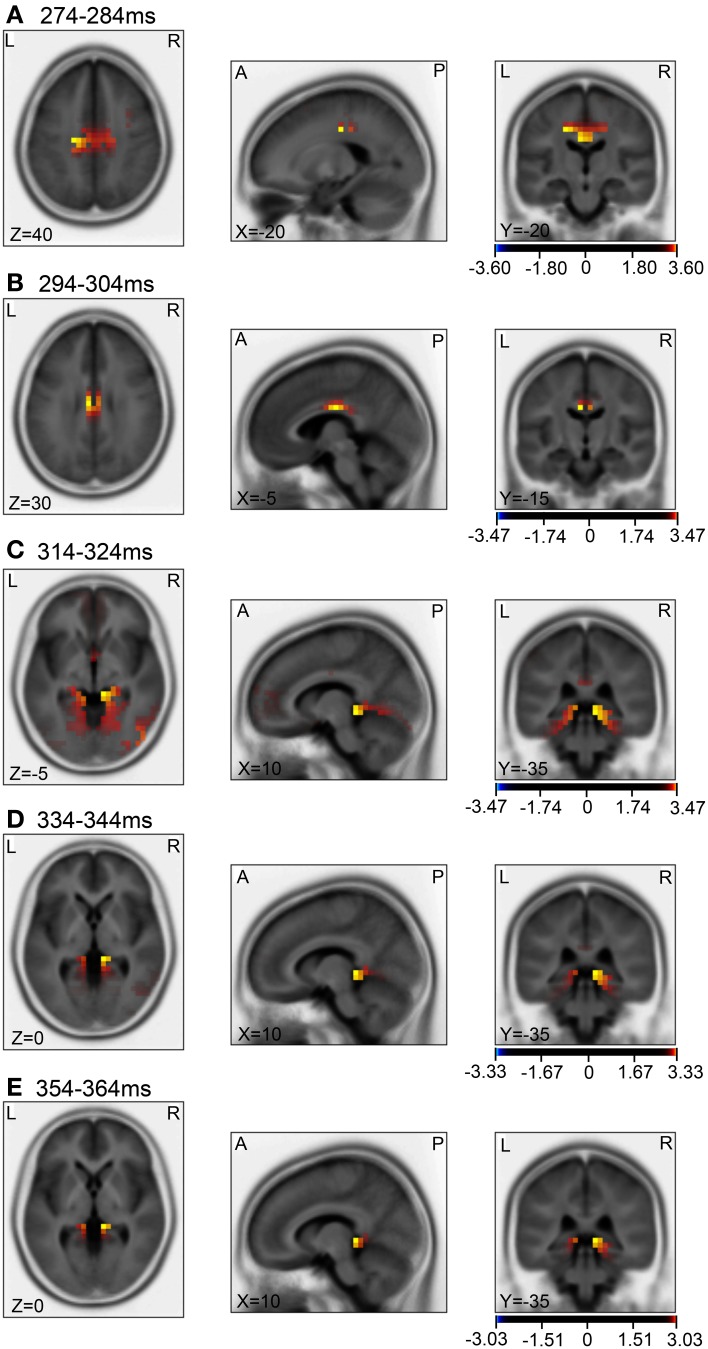
**sLORETA statistical nonparametric maps comparing the current source density between the ERPs in the baseline and the test phase in the 5 time windows, 274–284 ms (A), 294–304 ms (B), 314–324 ms (C), 334–344 ms (D), 354–364 ms (E), shown in Figure 9A**.

**Figure 11:**
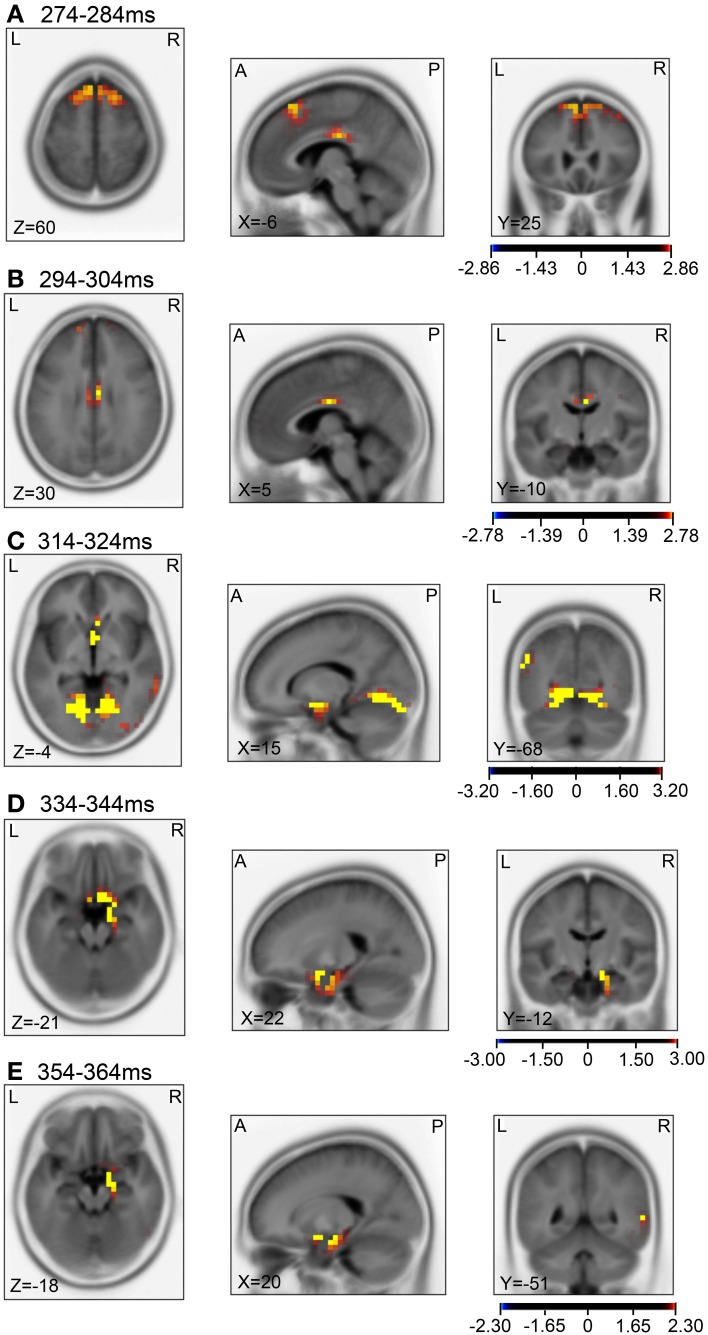
**sLORETA statistical nonparametric maps comparing the current source density of the ERPs between the control and test phases in the 5 time windows, 274–284 ms (A), 294–304 ms (B), 314–324 ms (C), 334–344 ms (D), 354–364 ms (E), shown in Figure 9B**.

Then, we analyzed the ERPs in every other 10-ms range around the peak in the same way. Figure 9 shows five 10-ms time windows subjected to sLORETA analysis in order to compare the current source density of the ERPs in the test phase to that of the ERPs at baseline **(A)** and in the control phase **(B)**. Figure 10 illustrates the brain areas with significant increases in current source density relative to baseline activity in the test phase. At latencies ranging from 274 to 284 and 294 to 304 ms, current source density was significantly higher in the posterior cingulate gyrus **(A,B)**. At the latencies ranging from 314 to 324, 334 to 344, and 354 to 364 ms, current source density was significantly higher in the entorhinal cortex/hippocampus, parahippocampal cortex, and lingual and fusiform gyri **(C–E)**. Figure 11 illustrates the brain regions in which significant increases in current source density were observed, in comparison with the control phase. At latencies from 274 to 284, and 294 to 304 ms, current source density was significantly higher in the superior frontal gyrus, including the medial prefrontal cortex **(A)** and the posterior cingulate cortex **(A,B)**. At the latencies between 314 and 324, 334 and 344, and 354 and 364 ms, current source density was significantly higher in the entorhinal and parahippocampal cortices, and lingual and fusiform gyri **(C–E)**. Furthermore, at the latencies from 314 to 324 and 354 to 364 ms, current source density was significantly higher in the left inferior parietal lobule **(C)** and the right posterior middle and inferior temporal cortex **(E)**, respectively.

## Discussion

### Evoked potentials for goal arrival and updating

In the present study, vertex-positive ERPs were elicited when the subjects entered the spatial reference points. Although the beep sound was presented upon arrival, these vertex-positive potentials were not just sensory evoked potentials. First, peak latencies of the vertex-positive potentials were relatively longer (more than 300 ms) than usual auditory evoked potentials. Second, amplitudes of the vertex-positive ERPs were larger in the test phase than in the control phase although the same beep sound was presented. The difference in cognitive demand between the control and test phases is that the subjects were not required to update their own location in the virtual town in the control phase, whereas the subjects in the test phase were required to update their own locations and to determine the direction of movement toward the next reference points. It has been reported that spatial updating is an automatic (involuntary) cognitive process, which is difficult to suppress (Farrell and Robertson, 1998; Farrell and Thomson, 1998). Consistent with this, the time required to solve the task was significantly increased in the test phase, suggesting that cognitive demand was larger in the test relative to the control phase. These findings suggest that the ERPs recorded in the present study reflect elevated cognitive processes recruited in spatial updating and in action planning for joystick manipulation while navigating successive reference points in space with no visible guides. Furthermore, these vertex-positive ERPs were not novelty-induced potentials (i.e., novelty P3) (Friedman et al., 2001; Ranganath and Rainier, 2003). In the present study, there were no significant differences in the vertex-positive ERP amplitudes between the first and third trials in the test phase; although the beep sound was repeatedly presented upon arrival at the reference points in the test phase, the amplitudes of the ERPs did not change over time. These findings also suggest that the vertex-positive ERPs reflected cognitive processes involved in spatial updating and action planning rather than stimulus novelty. Thus, the present study provides the first report of ERPs associated with spatial updating.

### Current source density analyses of the arrival-induced ERPs for long duration

Compared with the baseline before arrival, current source densities of the initial negative potentials in the latency ranging from 128–208 ms were significantly higher in the retrosplenial cortex and posterior insular cortex. Previous fMRI studies reported that scene images consistently activated the retrosplenial cortex (O'Craven and Kanwisher, 2000; Park et al., 2007). Since the retrosplenial cortex responded more strongly to scene images of familiar locations, this region might be involved in retrieval of scene memory (Epstein et al., 2007a,b). Furthermore, retrosplenial lesions in humans induce topographical amnesia, in which patients are unable to use landmarks to orient themselves (Aguirre and D'Esposito, 1999; Maguire, 2001; Epstein, 2008). Rodent neurophysiological studies reported that retrosplenial neurons (head direction cells) encode head direction (Chen et al., 1994; Cho and Sharp, 2001). These findings suggest that the retrosplenial cortex is involved in guiding navigation based on scene memory. On the other hand, previous fMRI studies reported that the posterior insula cortex encodes sense of self-motion in response to optical flow (Cardin and Smith, 2010), and this area was shown to be activated during mental navigation along memorized routes (Ghaem et al., 1997).

Compared with the baseline before arrival, the current source densities of the vertex-positive ERPs in the 274- to 374-ms latency were significantly higher in the posterior cingulate cortex. Consistent with the present results, previous human noninvasive studies also reported an increase in activity in the posterior cingulate cortex during virtual navigation (Grön et al., 2000; Pine et al., 2002) and during recall of known routes (Ghaem et al., 1997; Maguire et al., 1997). Furthermore, the cingulate sulcus in the posterior cingulate cortex has been reported to be involved in sense of self-motion in response to optical flow (Wall and Smith, 2008; Cardin and Smith, 2010). In addition, a monkey neurophysiological study reported that posterior cingulate cortical neurons encoded spatial locations in an allocentric reference frame (Dean and Platt, 2006). Taken together, the contrast between the baseline and reference point ERPs indicated that the brain regions involved in perception and recognition of sensory inputs during navigation (optical flow, familiar scenes) and those involved in guiding navigation, were activated.

Compared with the control phase, the current source density of the vertex-positive ERPs in the 274- to 374-ms latency was significantly higher in the superior frontal gyrus, including the medial frontal cortex (pre-SMA), entorhinal, and parahippocampal cortices. The superior frontal gyrus including the pre-SMA is activated during virtual driving (Spiers and Maguire, 2007b), and might be involved in monitoring traffic load and action planning during navigation (Spiers and Maguire, 2007b) since the pre-SMA has been implicated in performing planned voluntary movements (Lee et al., 1999; Cunnington et al., 2002; Lau et al., 2004). Interestingly, the most anterior part of the medial frontal cortex in the present study roughly corresponds to the area activated during virtual driving, which is an activation that also correlates with goal proximity (distance between current location and the goal) (i.e., distance between the present location and reference points in the present study) (Spiers and Maguire, 2007a). Furthermore, a human fMRI study reported that activity in the superior frontal gyrus is negatively correlated with random pointing errors in a virtual path integration task, suggesting that this brain region is involved in spatial working memory (Wolbers et al., 2007). On the other hand, the entorhinal cortex is also implicated in spatial navigation; a recent human fMRI study reported that characteristics of medial temporal cortical activity during virtual navigation suggested the existence of grid cells in the human entorhinal cortex (Doeller et al., 2010), and a neurophysiological study reported grid cells in the human entorhinal and cingulate cortices that were comparable to rodent grid cells (Jacobs et al., 2013). Furthermore, previous virtual navigation studies reported that the parahippocampal gyrus contains a region in its posterior extent called the parahippocampal place area, which shows increased activity in response to scenes, such as photographs of landscapes (Epstein et al., 2003). Neurophysiological studies reported that monkey and human parahippocampal neurons displayed place-related activities (Matsumura et al., 1999; Furuya et al., 2014), and also responded to specific landmarks in a viewpoint-dependent manner (Ekstrom et al., 2003; Weniger et al., 2010; Furuya et al., 2014). These findings along with human noninvasive studies (Epstein, 2008) suggest that the parahippocampal gyrus processes spatial information in egocentric or viewpoint-specific coordinates. Thus, the results in the present study, along with previous findings, suggest that arrival at the spatial reference points and subsequent spatial updating activate (1) brain regions involved in goal proximity and action planning and (2) brain regions involved in place recognition based on spatial information, including landmarks.

### Current source density analyses of the arrival-induced ERPs for short duration

Compared with the baseline (Figures 10A,B), current source densities of the ERPs in the 274- to 284- and the 294- to 304-ms latencies were significantly higher in the posterior cingulate cortex. The posterior cingulate cortex has been implicated in recalling known routes and sense of self-motion in response to optical flow (see above). Furthermore, current source density of the ERPs in the 314- to 324-, the 334- to 344-, and the 354- to 364-ms latencies were significantly higher in the entorhinal cortex/hippocampus, parahippocampal cortex, and lingual and fusiform gyri, compared to baseline (Figures 10C–E). The parahippocampal cortex is implicated in spatial function (see above). The fusiform and lingual gyri were reported to be activated during retrieval of spatial memory as well as during virtual navigation (Ekstrom and Bookheimer, 2007; Barra et al., 2012). Furthermore, landmark agnosia has been associated with lesions of the lingual gyrus (Aguirre and D'Esposito, 1999). Although the sLORETA does not provide conclusive information for the hippocampus due to inverse problems of source localization, this region also seemed to be activated in the present study. Other studies also reported hippocampal activation by LORETA (Cannon et al., 2005; Miyanishi et al., 2013). It has been reported that activity in the human hippocampus increases during spatial tasks performed in both real and virtual environments (Aguirre et al., 1996; Maguire et al., 1998), and damage to the hippocampus produces severe deficits in memory tasks performed in a real or virtual space in monkeys and humans (Astur et al., 2002; Hampton et al., 2004). The findings in these studies are consistent with those of a cognitive map theory in which the hippocampus acts as a cognitive map of the environment with allocentric coordinates (O'Keefe and Nadel, 1978). Consistent with this theory, the activities of some hippocampal neurons (place cells) increase in monkeys or humans when they navigate within a particular place in the environment in real and virtual navigation tasks (Nishijo et al., 1997; Matsumura et al., 1999; Ekstrom et al., 2003; Hori et al., 2005; Furuya et al., 2014).

Compared with the control phase (Figures 11A,B), current source densities of the ERPs in the 274- to 374- and the 294- to 304-ms latencies were significantly higher in the superior frontal gyrus including the medial prefrontal cortex and the posterior cingulate cortex (for discussion see above). Current source density of the ERPs in the 314- to 324- and the 334- to 344-ms latencies were significantly higher in the entorhinal cortex, parahippocampal cortex, and lingual and fusiform gyri, compared with the control phase (Figures 11C–E). The entorhinal cortex, parahippocampal cortex, and lingual and fusiform gyri have been implicated in navigation and landmark recognition (see above). Furthermore, current source densities of the ERPs in the 314- to 324- and the 354- to 364-ms latencies were significantly higher in the left inferior parietal lobule (Figure 11C) and right middle and inferior temporal cortex (Figure 11E), respectively. The inferior parietal lobule including its left side has been implicated in spatial attention and navigation accuracy (Maguire et al., 1998; Lee et al., 2013). Previous noninvasive studies reported that the right middle and inferior temporal cortex were activated during visual imagery of landmarks, and during encoding and recall of spatial relationships with objects (Ghaem et al., 1997; Johnsrude et al., 1999).

Thus, the short duration analyses indicated that similar brain regions to those in the long duration analyses were activated, and confirmed the results in the long duration analysis. Furthermore, it is noted that short duration analysis allows investigation of activation sequences. In both the comparisons, the brain regions involved in sensory perception and recall (posterior cingulate cortex involved in sense of self-motion, fusiform and lingual gyri involved in visual information processing) or evaluation of present location (medial prefrontal cortex) were initially activated (Figures 10A,B, 11A–C). In the later phase of the vertex-positive potentials, the parahippocampal gyri including the entorhinal and parahippocampal cortices as well as the hippocampus were activated (Figures 10C–E, 11D,E). These results suggest that the medial temporal lobe including these brain regions might receive all available information from other brain regions for spatial updating.

## Conclusions

The present study indicated that arrival at the spatial reference points and subsequent spatial updating elicited vertex-positive ERPs. Current source density analysis of the ERPs indicated that multiple parallel neural systems were active during spatial updating. Humans navigate their environment by dynamically updating spatial relations between their bodies and important landmarks in the surrounding environment using an egocentric system (Wang and Spelke, 2002). This dynamic egocentric system includes a path integration subsystem and a view (familiar landmarks)-dependent place recognition subsystem (Wang and Spelke, 2002). The present study indicated that these 2 subsystems were activated; the posterior cingulate cortex and posterior insular cortex in self-motion sensation during path integration, and the parahippocampal cortex in a viewpoint-dependent system for landmark-dependent place recognition. A human behavioral study suggests that these two subsystems interact and their information is integrated (Kalia et al., 2013). Furthermore, behavioral studies suggest that the egocentric system and allocentric system work in parallel during spatial updating and navigation (Burgess, 2006; Harvey et al., 2008). The present results indicate a parallel activation of allocentric (hippocampus) and egocentric (parahippocampal gyrus) systems. Our results provide neurophysiological evidence that humans use multiple spatial representations with different reference frames for spatial updating during navigation.

On the other hand, the inferior medial occipital lobe (lingual and fusiform gyri), right inferior temporal cortex, parahippocampal cortex, and hippocampus, which were activated during updating in the present study, are associated with route learning in a real environment (Barrash et al., 2000). The medial occipito-temporal cortices (lingual and fusiform gyri) and right inferior temporal cortex might be associated with the ability to quickly and accurately perceive and learn multiple topographical scenes, while the posterior parahippocampal gyrus and hippocampus might be involved in forming an integrated representation of the extended topographical environment (i.e., the appearance of places and spatial relationships between specific places), and consolidating that representation (Barrash et al., 2000). Compared with the previous studies that investigated remote spatial memory, which is established for many years (see a review by Spiers and Maguire, 2007c), the present experiments imposed only six trials including both the control and test trials at one time. These findings suggest that not only updating processes but also learning and consolidation processes take place simultaneously.

Finally, it is noted that human subjects display individual differences in navigation strategies [e.g., allocentric (bird-view) or egocentric (landmark) strategies] (Jordan et al., 2004). In the present study, we could not classify the subjects based on their navigation strategies since they were required to navigate in the fixed route to receive the same visual stimuli in the virtual space. Further studies are required to investigate brain activation differences based on navigation strategies. The present study at least indicated common neural networks among the subjects during spatial updating.

## Author contributions

Hisao Nishijo designed the research; Hai M. Nguyen, Jumpei Matsumoto, and Hisao Nishijo performed research; Hai M. Nguyen, Jumpei Matsumoto, Anh H. Tran, Taketoshi Ono, and Hisao Nishijo analyzed data; and Hai M. Nguyen and Hisao Nishijo wrote the paper.

### Conflict of interest statement

The authors declare that the research was conducted in the absence of any commercial or financial relationships that could be construed as a potential conflict of interest.
